# Dysphagia and Tongue Deviation: A Rare Case of Collett–Sicard Syndrome after Blunt Head Trauma

**DOI:** 10.3390/neurolint12030019

**Published:** 2020-12-21

**Authors:** Eric Tamrazian, Bijal Mehta

**Affiliations:** 1 Department of Neurology, David Geffen School of Medicine, Harbor-UCLA Medical Center, Torrance, CA 90502, USA; tamrazianeric@gmail.com; 2 Los Angeles Biomedical Institute, Los Angeles, CA 90095, USA

**Keywords:** dysphagia, tongue deviation, subarachnoid hemorrhage

## Abstract

The jugular foramen and the hypoglossal canal are both apertures located at the base of the skull. Multiple lower cranial nerve palsies tend to occur with injuries to these structures. The pattern of injuries tend to correlate with the combination of nerves damaged. Case Report: A 28-year-old male was involved in an AVP injury while crossing the highway. Exam showed a GCS of 15 AAOx3, with dysphagia, tongue deviation to the right, uvula deviation to the left and a depressed palate. Initial imaging showed B/L frontal traumatic Sub-Arachnoid Hemorrhages (tSAH), Left Frontal Epidural Hematoma and a Basilar Skull Fracture. On second look by a trained Neuroradiologist c At 3 month follow up, patient’s tongue normalized to midline and his dysphagia resolved. Discussion: Collette-Sicard syndrome is a rare condition/syndrome characterized by unilateral palsy of CN: IX, X, XII. This condition has been rarely described as a consequence of blunt head trauma. In most cases, the condition is self-limiting with patients regaining most to all of their neurological functions within 6 months. Nerve traction injuries and soft tissue edema compressing the cranial nerves are the leading two hypothesis. In conclusion, injuries with focal neurological deficits which were not apparent on initial imaging should be reviewed by relevant experts with concomitant knowledge of the patient’s history.

## 1. Introduction

Collet–Sicard Syndrome (CSS) is a rare condition characterized by unilateral palsy of CN: IX, X, XI and XII first described in soldiers during World War I, by Dr. Collet in 1915 and later by Dr. Sicard [1,2]. This condition was historically attributed to tumors of the skull base, coiling and dissections of the internal carotid artery, multiple myeloma, vasculitis, carotid fibromuscular dysplasia, shotgun injuries, idiopathic cranial polyneuropathy, atlas fractures, and occipital condyle fractures [3,4,5]. The jugular foramen and the hypoglossal canal are both apertures located at the base of the skull. The jugular foramen contains the cranial nerves, IX, X and XI, involved in normal cough and gag reflex. The hypoglossal canal contains the cranial nerve XII, responsible for movements of the tongue. Therefore, multiple lower cranial nerve palsies tend to occur with injuries to these structures. The pattern of injuries tends to correlate with the combination of nerves damaged. Presentation of CSS from blunt head trauma is very rare and has only been reported a few times. Therefore, no management guidelines exist for the treatment of such cases. Here, we report a rare case of CSS with dysphagia and tongue deviation from a blunt head trauma.

## 2. Case Report

A 28-year-old male was involved in an auto-vs.-pedestrian injury while crossing the highway. The patient was brought to Harbor-UCLA Medical Center, a level-I trauma center. On admission, he was complaining of a headache and an inability to swallow his own saliva. Exam showed a GCS of 15 AAOx3, with dysphagia, tongue deviation to the right, uvula deviation to the left and a depressed palate. No other abnormal findings were noted. Initial imaging showed bilateral frontal traumatic subarachnoid hemorrhage (tSAH), left frontal epidural hematoma (EDH) and a basilar skull fracture. The patient was admitted to the ICU with neuro-checks every hour. The patient remained neurologically stable. On hospital day 4, he was transferred out of the ICU with persistent tongue deviation and an inability to swallow. Speech pathology was consulted and speech therapy initiated with no improvement of his dysphagia. Initial radiology read by a radiologist did not reveal any further structural abnormalities then the ones stated above. Neurology was consulted and imaging was reviewed by a neuroimaging-trained neurologist, which demonstrated injury to the wall of the jugular foramen and the hypoglossal canal. Nasogastric tube feeding was initiated and the patient had a Percutaneous Endoscopic Gastrostomy (PEG) tube placed on hospital day 17 and was discharged home. At 3 month follow-up, the patient’s tongue normalized to midline and his dysphagia resolved. His PEG tube was removed at the 3 month follow-up. As this was a retrospective study and patient identifiers were not used, written consent was not obtained. However, on clinic follow up, verbal consent was obtained which the patient agreed.

## 3. Discussion

**Figure 1:**
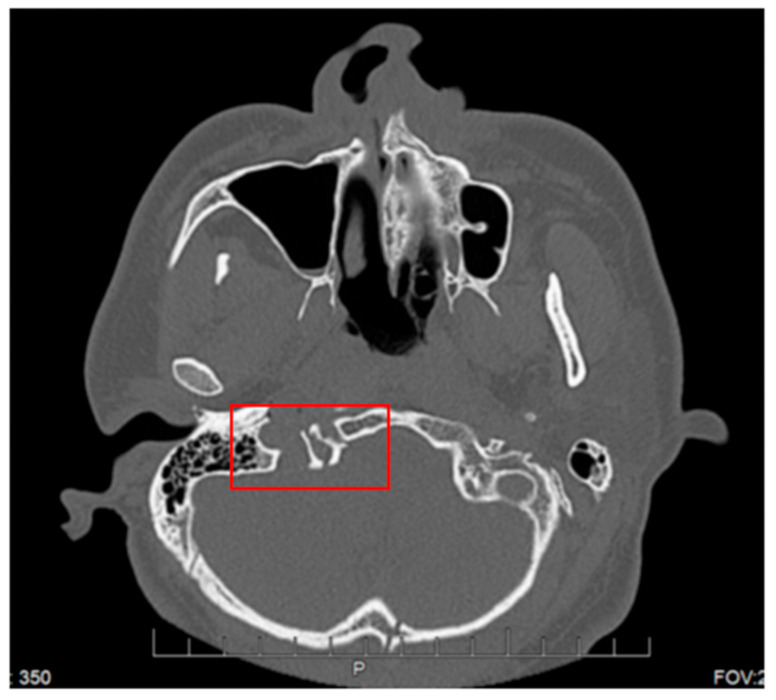
CT maxillofacial. Red box indicates junction of Jugular foramen and Hypoglossal Canal.

**Figure 2:**
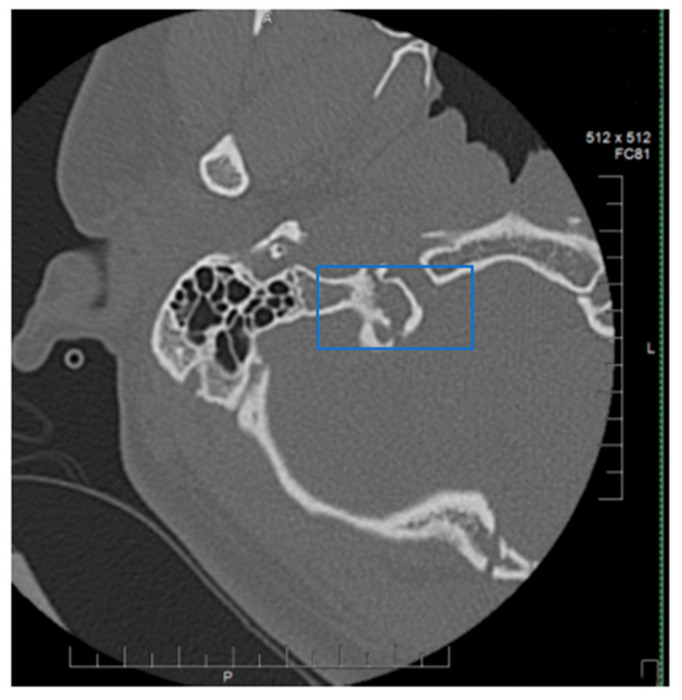
CT temporal bone. Highlighted blue box indicates junction of Jugular foramen and Hypoglossal Canal.

**Figure 3:**
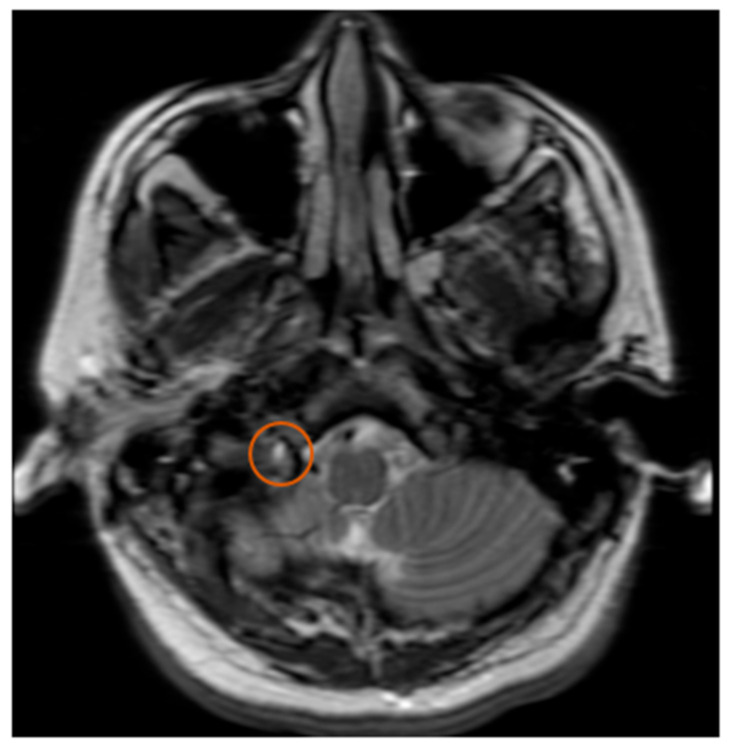
MRI T2. Orange circle indicates edema at the junction of the Jugular Foramen and the Hypoglossal Canal.

Collet–Sicard Syndrome has rarely been described as a consequence of blunt head trauma. Injuries to the jugular foramen and the hypoglossal canal are rare, as most blunt head traumas resulting in basilar skull fracture involve the condyles. In most cases, the condition is self-limiting, with patients regaining most to all of their neurological functions within 6 months. Several theories have been proposed for the pathophysiology of this syndrome. Displaced bony fragments that could compress the nerves in the context of fracture extension to the posterior jugular foramen or to the hypoglossal canal has been one theory to explain the neurological manifestations seen with CSS [6]. Bridgman et al. suggested that the cranial nerves were damaged by traction injuries [7]. Orbay et al. reported a case in which hypoglossal nerve palsy occurred 3 months after a head injury and suggested that a scarring process and the formation of a callus at the level of the hypoglossal canal could progressively compress the main hypoglossal nerve [8]. One of the more recent theories is that soft tissue edema, as a consequence of the local injury, compresses the cranial nerves (9, 10, 11 and 12) and leads to their deficits. With time, as the edema resolves, the neurological deficits also resolve [9]. The initial fracture, as can be seen by Figure 1, demonstrates a fracture on the border between the jugular foramen and the hypoglossal canal. CT of the Temporal bone better demonstrates the fracture location (See Figure 2). A subsequent MRI of the brain also demonstrates an area of hypo-attenuation, consistent with edema at the location of the jugular foramen and the hypoglossal canal (Figure 3). Treatment for CSS with blunt head trauma remains conservative, as other causes, such as a tumor or vascular impingement, require treatment of the underlying cause. As the edema resolves, so do neurological deficits. Some authors have advocated the anecdotal use of steroids to reduce the edema; however, as this case is very rare, large multi-institutional trials are warranted to better elucidate the role of steroids with Collet–Sicard syndrome. Of important note is to readily identify this condition and provide nutritional support with feeding tubes, as the recovery time is generally 3–6 months. In conclusion, injuries with focal neurological deficits which were not apparent on initial imaging should be reviewed by relevant experts with concomitant knowledge of the patient’s history and experience in neuroimaging.
